# Fatty Acid Synthase-Derived Lipid Stores Support Breast Cancer Metastasis

**DOI:** 10.21203/rs.3.rs-5510550/v1

**Published:** 2024-12-05

**Authors:** Chaylen Andolino, Eylem Kulkoyluoglu Cotul, Zilin Xianyu, Yun Li, Divya Bhat, Mitchell Ayers, Kimberly K. Buhman, Stephen D. Hursting, Michael K. Wendt, Dorothy Teegarden

**Affiliations:** Purdue University West Lafayette; Purdue University West Lafayette; Purdue University West Lafayette; University of Iowa; University of Iowa; Purdue University West Lafayette; Purdue University West Lafayette; University of North Carolina at Chapel Hill; University of Iowa; Purdue University West Lafayette

**Keywords:** fatty acid synthase, FASN, breast cancer, TNBC, lipid droplet, lipid metabolism, metastasis, lipid storage, fatty acids, mass spectrometry

## Abstract

Lipid accumulation is associated with breast cancer metastasis. However, the mechanisms underlying how breast cancer cells increase lipid stores and their functional role in disease progression remain incompletely understood. Herein we quantified changes in lipid metabolism and characterized cytoplasmic lipid droplets in metastatic versus non-metastatic breast cancer cells. ^14^C-labeled palmitate was used to determine differences in fatty acid (FA) uptake and oxidation. Despite similar levels of palmitate uptake, metastatic cells increase lipid accumulation and oxidation of endogenous FAs compared to non-metastatic cells. Isotope tracing also demonstrated that metastatic cells support increased *de novo* lipogenesis by converting higher levels of glutamine and glucose into the FA precursor, citrate. Consistent with this, metastatic cells displayed increased levels of fatty acid synthase (FASN) and *de novo* lipogenesis. Genetic depletion or pharmacologic inhibition of FASN reduced cell migration, survival in anoikis assays, and *in vivo* metastasis. Finally, global proteomic analysis indicated that proteins involved in proteasome function, mitotic cell cycle, and intracellular protein transport were reduced following FASN inhibition of metastatic cells. Overall, these studies demonstrate that breast cancer metastases accumulate FAs by increasing *de novo* lipogenesis, storing TAG as cytoplasmic lipid droplets, and catabolizing these stores to drive several FAO-dependent steps in metastasis.

## Introduction

Breast cancer is the second leading cause of cancer-related deaths in women (1). Progression to metastatic disease accounts for the majority of patient mortality; therefore, preventing progression and treating metastases is necessary for alleviating the burden of this devastating disease. The metastatic cascade is comprised of steps including migration, dissemination, adaptation to new microenvironment, and growth at the secondary site (2, 3). Cancer cells adapt to new environments in part by their dysregulated energy metabolism, which includes the utilization of different metabolic substrates to successfully colonize distant organs (4). Metabolic plasticity is emerging as a critical process that promotes cell survival during stress through utilization of different substrates, such as glucose, glutamine, and fatty acids (FA) (5–7).

Though many cancer metabolism studies have focused on glucose and glutamine metabolism (8–10), the contribution of lipids has emerged as an area of investigation, particularly given that metabolic pathways and substrates utilized by cancer cells for necessary functions, such as proliferation, growth, migration, and oxidative stress protection converge at FAs. Glucose and glutamine are utilized for FA synthesis following entry into the tricarboxylic acid (TCA) cycle and exit into the cytoplasm as citrate (14). Citrate and coenzyme A are converted to acetyl-CoA by ATP citrate lyase (ACLY), which is then metabolized by acetyl-CoA carboxylase (ACC) to form malonyl-CoA and FA synthase (FASN) to generate palmitate (15). Thus, non-lipid substrates are metabolized to support the synthesis of endogenous FAs—a pathway that has been implicated in breast cancer progression (16–18). FASN is upregulated in many cancers, including the aggressive triple negative breast cancer (TNBC) subtype (19–21). In addition, increased uptake of FAs compared to non-malignant tissue has also been observed in TNBC (22). Thus, both endogenous and exogenous FA sources may contribute to neutral lipid stores in aggressive cancer types.

One consequence of dysregulated FA metabolism pathways in breast cancer is the accumulation of lipids within these cells, which provides a reservoir of FAs to serve cancer-promoting functions. These lipid stores, termed lipid droplets (LDs), are associated with treatment resistance and increased likelihood of relapse (23–25). Several hypotheses exist regarding how LDs may promote cancer progression, such as protection against lipotoxicity, provision of FAs for membrane biosynthesis or energy production, regulation of autophagy, and mediation of ER stress homeostasis (26). Additionally, LD biogenesis is known to occur during times of stress, such as in low oxygen environments (hypoxia), chemotherapy, oxidative stress, and nutrient deprivation (27, 28).

In the current study, we investigated the mechanisms by which metastatic breast cancer cells acquire LDs and what role lipid metabolism plays in migration, survival in detached conditions, and overall completion of metastasis. We sought to address the hypothesis that FA synthesis contributes to LD stores that characterize metastatic breast cancer cells compared to their non-metastatic counterparts, and that production and utilization of these stores is required to complete metastatic processes. We identify a key role of FASN in promoting lipid accumulation and metastatic progression utilizing *in vitro*, *ex vivo*, and *in vivo* model systems in combination with genetic manipulation and emerging therapeutic strategies.

## Methods

### Cell culture

MCF10A-*ras* and MCF10CA1a cells from the same cell lineage, which form primary tumors that metastasize to the lungs *in vivo*, were cultured as previously described (29–32). Normal murine mammary gland cells transformed by EGFR overexpression (NME) cells and their lung metastatic derivatives (LM2) were constructed and cultured as previously described (33, 34). The 4T1 cell line was purchased from ATCC and modified to stably express firefly luciferase under Zeocin selection (35). Lung metastases were obtained from the lungs of 4T1 tumor-bearing mice. The lungs were mechanically disrupted, incubated in 5% trypsin/EDTA and plated in the presence of Zeocin. *Ex vivo* cultures were analyzed within four passages post-isolation.

Small molecule inhibitors used for experiments are as follows: 75 μM etomoxir (CPT1 inhibitor; E1905, Sigma), 0.01–20 μM TVB-3166 (FASN inhibitor; SML1694, Sigma), 50 μM PF 04620110 (DGAT1 inhibitor; PZ0207, Sigma), and 10 μM PF 06424439 (DGAT2 inhibitor; PZ0233, Sigma). All vehicle-treated cells contained the greatest volume of vehicle solvent utilized for the inhibitors, where dimethyl sulfoxide (DMSO) remained under 0.06% of total volume.

### Transmission electron microscopy

Cells were fixed in 2.5% glutaraldehyde in 0.1 M sodium cacodylate buffer, rinsed, and embedded in agarose. The cell pellet was fixed in 1% osmium tetroxide containing 0.8% potassium ferricyanide and stained in 1% uranyl acetate and prepared as previously described (36). For quantification, 50 cells from each model were analyzed for LD analysis by ImageJ.

### Oil red O staining

Lipid levels were assessed via Oil Red O (Sigma-Aldrich, MAK194) staining following PBS washing, 4% paraformaldehyde fixation, and 60% isopropanol rinse as per manufacturer’s instructions.

### Triacylglycerol (TAG) assay

TAG was quantified using a colorimetric assay according to the manufacturer’s instruction (Wako L-Type Triglyceride M Kit, Wako Diagnostics USA). Absorbance was measured at 600 nm and results were normalized to protein content (31, 36). In experiments with inhibitors as pretreatments, cells were treated for 72 hours with either DMSO (vehicle), 20 μM TVB-3166 (FASN inhibitor), or PF 04620110 (50 μM) and PF 06424439 (10 μM) to inhibit both DGAT isozymes. Media with treatments were replaced every 24 hours.

### Wound healing migration assay

Cells were grown to 95% confluency, scored with a pipette tip, rinsed with 1X phosphate buffered saline (10X PBS Solution; Fisher) and replenished with 1% horse serum-containing media, containing either DMSO or 75 μM etomoxir. Images were acquired at 9, 18 and 24 hours at the same location within each well and TScratch software was used to calculate percent wound closure (CSE-Lab).

### Transwell migration assay

For pretreated experiments (as described for TAG assay), cells with confirmed TAG depletion were seeded into 8 μm pore transwells (Corning, Corning, NY) in serum-free media with either DMSO or 75 μM etomoxir and mounted into 24 well plates for 18 hours. Serum-containing media was added below the transwells. Migration was assessed following methanol fixation and crystal violet staining. Cells were counted from photos of five random fields of each transwell. The proportion of migrated cells compared to the total cell count (non-migrated plus migrated cells) was assessed. For the migration assay with ATGL (ATGListatin, 20 μM) and CPT1a (etomoxir, 75 μM) inhibition, cells were treated only during the 18-hour incubation in the transwell.

### ^14^ C-Palmitate uptake and oxidation assays

For FA uptake assays, cells were grown to 80% confluency in 35 mm dishes. Immediately prior to beginning the experiment, cells were replenished with fresh media without additional growth factors standardly supplemented in the MCF10A-*ras* media. Cells or empty dishes (blank) were given media containing 1 mM palmitate, including 1,000,000 disintegrations per minute (DPM) ^14^C-palmitate conjugated to 1% BSA. Following incubation for 10 minutes at 37°C, cells were rinsed twice with PBS, scraped into 1 mL PBS and collected for analysis via liquid scintillation counting. Three additional dishes were plated and treated as described above and utilized to normalize samples to protein concentration determined by BCA assay.

Catabolism of an external FA source was determined by assessment of radiolabeled CO_2_ released following ^14^C-palmitate oxidation within a closed chamber, as previously described (37). Samples were incubated for 1 hour at room temperature and radioactivity of the captured ^14^CO_2_ in center well’s filter paper was determined. Results are expressed as nmol of ^14^CO_2_ captured per total protein.

### Endogenous fatty acid oxidation (Seahorse) assay

Fatty acid oxidation (FAO) from internal stores was determined with an XFe24 Well Seahorse Analyzer (102340–100, Agilent). Cells were given media containing 17.5 mM glucose, 2.5 mM glutamine, and 0.5 mM pyruvate without serum for 1 hour and assayed according to the manufacturer’s instructions utilizing the Seahorse XF Substrate Oxidation Stress Test Kit for Long Chain Fatty Acid Oxidation (103672–100, Agilent) with 1 μM nal FCCP concentration. The sequential compound injections of oligomycin, FCCP, and rotenone/antimycin A measure acute response to etomoxir, and maximal respiration in the absence and presence of inhibitor. The average value from blank wells was subtracted from each experimental well, and each value was normalized to total protein as determined by BCA assay.

### RNA isolation and quantitative polymerase chain reaction (qPCR) analysis

MCF10A-derived cells were harvested and isolated using Tri-Reagent (Sigma, 93289) following the manufacturer’s instructions. Reverse transcription of RNA by MMLV reverse transcriptase (Promega, Madison, WI) and real-time quantitative PCR was completed using a LightCycler 480 instrument with SYBR Green I Master Mix (Roche, Indianapolis, IN). For 4T1 cells, RNA was extracted using Omega Biosciences’ kit and transcribed into cDNA with Thermo Fisher’s Verso kit. The resulting cDNA was quantified with qRT-PCR using Maxima SYBR Green/ROX qPCR Mastermix on a Bio-Rad CFX Connect Real-Time System. mRNA abundance of target genes (Table 1) was normalized to 18S or GAPDH abundance. Results represent arbitrary units as described previously (38).

### Immunoblotting

Cells were washed with ice-cold PBS prior to harvesting in radioimmunoprecipitation assay (RIPA) buffer (Cell Signaling, Danvers, MA) with 1% phenylmethanesulfonyl fluoride protease inhibitor (PMSF, Cell Signaling) and phosphatase inhibitor cocktail 2 (P5726, Sigma). Cells were scraped and lysed by freeze-thaw, sonication, and vortexing followed by centrifugation at 13,000 RPM for 15 min. Protein concentration of the supernatant was determined (BCA assay) and 20 μg was separated on 7.5% polyacrylamide gels and transferred to 0.2 μm nitrocellulose membrane (Bio-Rad, Hercules CA). Membranes were incubated with antibodies for Actin and FASN (C20G5, Cell Signaling) overnight in 5% non-fat dry milk and detected using an Odyssey CLx imaging system (Li-Cor, Lincoln, NE).

### Fatty acid synthesis and metabolic flux

For *de novo* lipogenesis analysis, MCF10CA1a cells were grown to 60% confluency and incubated with standard media containing additional ^13^C_2_-acetate (10 mM), or 100% ^13^C_6_-glucose (17.5 mM) or ^13^C_5_-glutamine (2.5 mM). After 24 hours, FAs were hydrolyzed by hydrochloric acid and extracted for analysis using Agilent 6460 Triple Quadrupole Liquid Chromatography–Mass Spectrometry (LC–MS/MS) (31). The primary FA *de novo* lipogenesis product, 16:0 palmitate, was quantified. Area under the curve (AUC) for the stable isotope-labeled palmitate product detected by LC–MS/MS was obtained based on retention time alignment to a known standard. Final values represent percent of total palmitate pool derived from ^13^C–substrate, calculated by (AUCfromC13FAsTotalAUCfromallFAs*100).

For metabolic flux analysis, cells were grown to 80% confluency and media was replaced with media containing 100% ^13^C_6_-glucose (17.5 mM) or 100% ^13^C_5_-glutamine (2.5 mM) for 2 hours. Metabolites were extracted and derivatized as previously described (32) and analyzed using gas chromatography-mass spectrometry with a TG-5MS gas chromatography column and Thermo TSQ 8000 Triple Quadrupole Mass Spectrometer. MS data was corrected using IsoCor software and pool sizes were calculated by dividing total metabolite AUC by norvaline AUC and mg protein to account for variations in metabolite recovery and total cell quantity, respectively.

### Extracellular matrix attachment viability assay

Extracellular matrix (ECM) detachment was simulated using poly 2-hydroxyethyl methacrylate (poly-HEMA) coated plates, prepared as previously described (32). Viability assays were performed according to the manufacturer’s (Sigma) instructions using 3–4,5-dimethylthiazol-2-yl-2,5-diphenyltetrazolium bromide (MTT) following incubation with vehicle (DMSO) or FASN inhibitor TVB-3166 (20 μM) for 72 hours. Media with treatments was replaced every 24 hours, followed by plating cells into poly-HEMA coated plates for 40 hours and analysis.

### Proteomic analysis

Cells were treated with DMSO or 20 μM TVB-3166 for 72 hours prior to harvesting for untargeted global proteomic analysis. An aliquot of 50 μg of these whole cell lysate (WCL) samples were delipidated, precipitated, and digested with Trypsin/Lys-C for bottom-up proteomics. Detailed description of sample preparation and LC-MS/MS parameters and analysis are as previously described (36).

### In vivo experiments

All animal-related research activities were performed in compliance with protocols sanctioned by Purdue University, adhering to NIH guidelines for animal usage and welfare, under the specific IACUC protocol numbered 1310000978. Tumor volume was calculated by $\frac{\text{length}*\text{widt}h^{2}}{2}$ . Balb/c mice were used for orthotopic mammary fat pad injections of the 4T1 cells. Control and FASN depleted cells (5×10^4^/50 μL) were transplanted into the fourth mammary fat pad and primary tumor growth was monitored every other day via caliper measurement. Ten days after the injection, tumors were removed, and pulmonary metastatic formation was monitored by bioluminescence imaging for 16 more days using an AMI imager (Spectral Instruments Imaging, Tucson, AR) as previously described (39).

### Lipid analysis by multiple reaction monitoring (MRM) pro ling

Multiple reaction monitoring (MRM) profiling was used to profile several classes of lipids from 4T1 primary tumors and lung metastases. Lipids were extracted by Bligh-Dyer and prepared as previously described (40, 41). Ten μL of diluted lipid extract was flow injected into the ion source of an Agilent 6410 QQQ mass spectrometer via am Agilent 1100 micro-autosampler. Statistical analysis and annotation were performed using Metaboanalyst 5.0 and LION/web (42).

### Statistical analyses

A two-tailed Student’s t-test was used for comparing the difference between two groups of data in *in vitro* assays. Error bars indicate the standard error of the mean. For *in vivo* experiments, the measurements were compared using a two-way ANOVA, multiple comparisons test. Analyses were performed via GraphPad Prism 10 software.

## Results

### Metastatic breast cancer cells accumulate lipid

**Figure 1:**
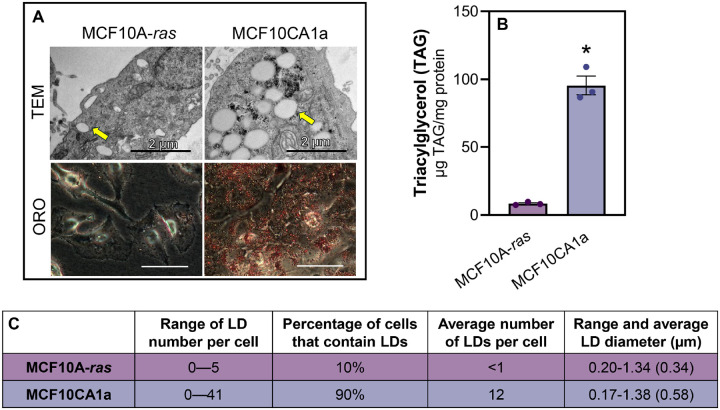
Metastatic breast cancer cells accumulate lipid. **A** Representative transmission electron micrographs of MCF10A-*ras* (top) or MCF10CA1a (bottom) cells. Images stacked above and below each other were taken at the same magnification. The yellow arrow indicates LDs. **B** Quantification of TEM imaging; size of LDs determined using ImageJ. **C** Representative Oil Red O images of MCF10A-ras(left) or MCF10CA1a (right) cells. Images were taken at the same magnification. **D** TAG accumulation in MCF10A-ras(white) and MCF10CA1a (gold) cells. Asterisk indicates p < 0.05 between MCF10A-*ras* and MCF10CA1a; 3 biological replicates per cell line.

To assess the level of lipid accumulation in metastatic compared to non-metastatic breast cancer cells, TAG as well as LD abundance and size were measured. Using transmission electron microscopy (TEM) and Oil Red O staining, we demonstrated that metastatic MCF10CA1a cells have significantly greater LD accumulation compared to their isogenic and non-metastatic MCF10A-*ras* counterpart (Fig. 1A–C). Consistent with these results, the majority (90%) of the metastatic cells contained at least one LD, whereas only 10% of the non-metastatic cells contained LDs (Fig. 1C). Although the range of LD sizes was similar, the average size of LDs in metastatic cells was larger than those within non-metastatic cells (Fig. 1C). Consistent with our previous results, non-metastatic cells have significantly less TAG accumulation compared to the metastatic cells (Fig. 1B) (31). Thus, metastatic cells contain higher levels of TAG as well as more abundant and larger LDs than the non-metastatic cells.

### Fatty acid oxidation is required for metastatic cell migration

**Figure 2:**
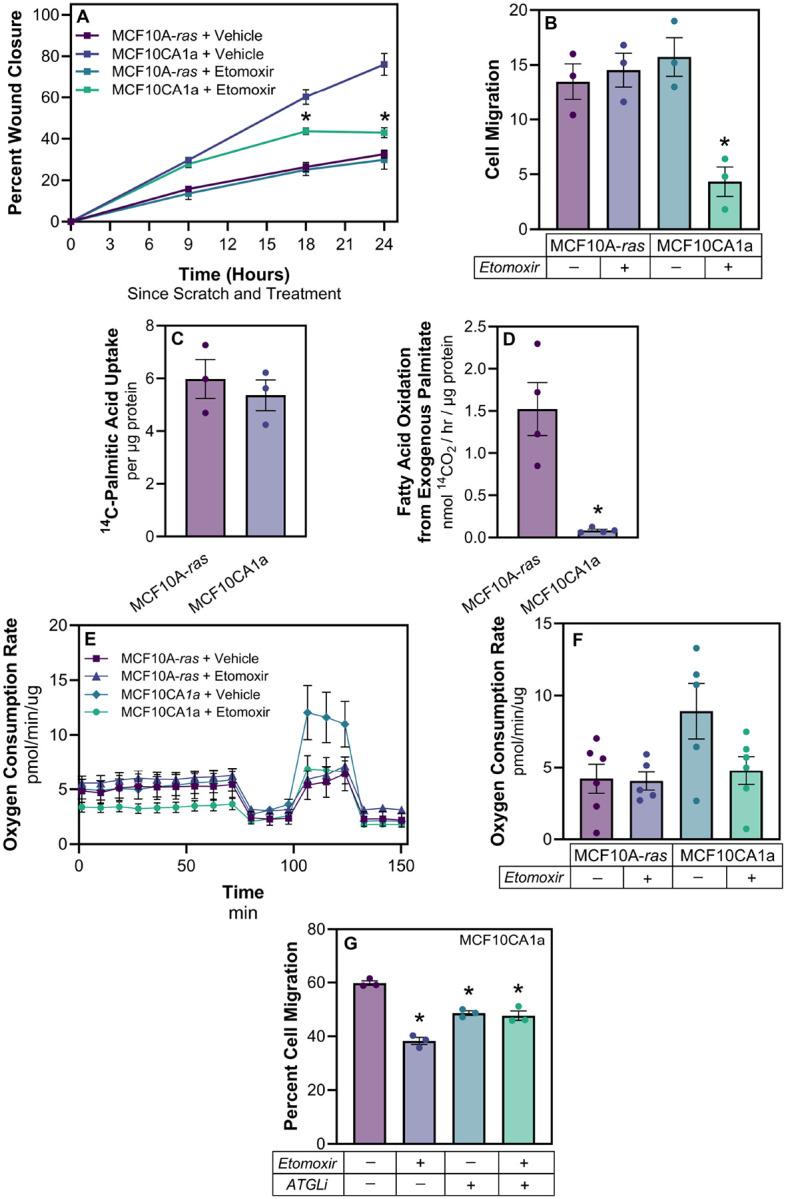
Fatty acid oxidation is required for metastatic cell migration. **A** Wound healing assay using MCF10A-*ras* (black) and MCF10CA1a (gold) cells, either treated with vehicle (DMSO; dotted) or etomoxir (75 μM; solid). **B** Transwell migration assay following 18 hours of treatment as described in (A). **C** ^14^Cpalmitate uptake in MCF10A-*ras* (white) and MCF10CA1a (gold) cells after 15 minutes of incubation. **D** ^14^C-palmitate oxidation in MCF10A-*ras* (white) and MCF10CA1a (gold) cells after 2 hours of incubation. **E** Oxygen consumption rate (OCR) in MCF10A-*ras* (darker shades) and MCF10CA1a (lighter shades) with either media (blue) or FAO inhibitor (etomoxir, green). Basal response is indicated as time before first injection, acute response to FAO inhibition is displayed following second injection, and maximal response to FAO inhibition is demonstrated following the third injection. **F** Histogram of maximal response in vehicle- and etomoxir-treated cells. **G** Cells were treated with either vehicle (DMSO, white), FAO inhibitor (etomoxir, gray), adipose triacylglycerol lipase inhibitor (ATGListatin; black check), or etomoxir and ATGListatin together (gray and black check) during 18 hour transwell incubation. Asterisks indicate p < 0.05 between MCF10A-*ras* and MCF10CA1a, or treatment and vehicle, at given time point (A) or at end of assay (B); 3 biological replicates per cell line or treatment; 6 technical replicates per Seahorse assay. Seahorse assay data presented is representative of results from two independent experiments.

Given the difference in lipid accumulation between the two cell lines, we hypothesized that metastatic cells may utilize FAs differently for key processes, such as migration. The migration of metastatic MCF10CA1a cells was significantly greater than non-metastatic MCF10A-*ras* cells (Fig. 2A). Cells treated with etomoxir, an inhibitor of CPT1, the rate-limiting enzyme necessary to import activated FAs into the mitochondria for oxidation, reduced the level of MCF10CA1a cell migration, as measured by a wound healing assay (Fig. 2A). In contrast, there was no effect on the MCF10A-*ras* cells (Fig. 2A). Similar results were observed using a transwell migration assay (Fig. 2B).

We also examined the utilization of exogenous and endogenous FAs. There was no significant difference between ^14^C-palmitate uptake between the MCF10A-*ras* and MCF10CA1a cells; however, the oxidation of exogenous ^14^C-palmitate was significantly greater in the non-metastatic MCF10A-*ras* cell line compared to the metastatic MCF10CA1a cells (Fig. 2C and D). Conversely, endogenous FA oxidation was significantly greater in the MCF10CA1a cells compared to MCF10A-*ras* (Fig. 2E). Following depletion of exogenous FAs in the media and inhibition of FAO with etomoxir, oxygen consumption rate (OCR) of the MCF10A-*ras* cells remained unaffected (Fig. 2F). In contrast, when MCF10CA1a cells were treated with etomoxir, OCR was reduced to the level of MCF10A-*ras* cells (Fig. 2E and F). Together, these results demonstrate that metastatic MCF10CA1a cells have greater oxidation of stored FAs, whereas non-metastatic MCF10A-*ras* cells catabolize exogenous FAs at a higher level than the MCF10CA1a cells.

To determine if FAO-dependent MCF10CA1a cell migration utilizes the TAG stores within the cells, both FAO and TAG lipolysis were simultaneously inhibited during a transwell migration assay (Fig. 2G). MCF10CA1a cells were treated with either vehicle, etomoxir, the adipose triacylglycerol lipase inhibitor (ATGListatin), or both. Consistent with the results from Fig. 2A and B, inhibiting FAO reduces MCF10CA1a cell migration. In addition, inhibiting the first step of the TAG lipolysis cascade, ATGL, also significantly reduced MCF10CA1a cell migration (Fig. 2G). Notably, when both FAO and TAG lipolysis were inhibited simultaneously, MCF10CA1a cell migration was not further reduced (Fig. 2G). Collectively, these results indicate that metastatic cells rely on lipolysis and that FAs derived from stored lipids sustain FAO-dependent cell migration.

### Metastatic cells have increased fatty acid synthesis from non-lipid substrates

**Figure 3:**
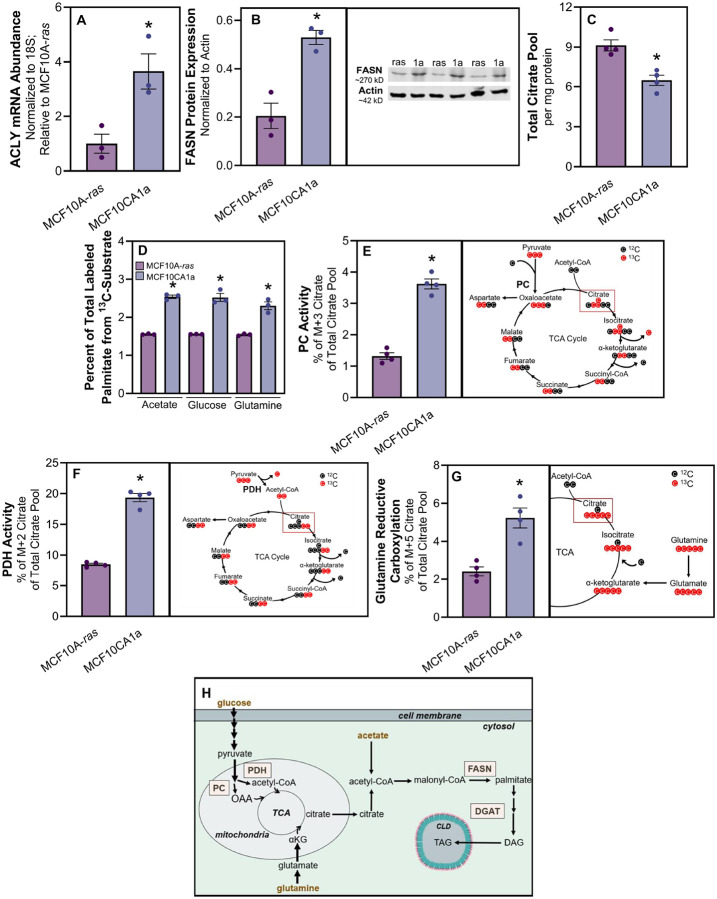
Metastatic cells have increased fatty acid synthesis from non-lipid substrates. **A** Relative ATP citrate lyase (ACLY) mRNA abundance in MCF10A-*ras* (white) and MCF10CA1a (gold) cells. **B** Fatty acid synthase (FASN) protein expression between MCF10A-*ras* (white) and MCF10CA1a (gold) cells. C Total citrate pool size between MCF10A-*ras* (white) and MCF10CA1a (gold) cells. D Percent of carbon incorporation from acetate, glucose, or glutamine into palmitate in MCF10A-*ras* (white) or MCF10CA1a (gold) cells. **E** Pyruvate carboxylase (PC) activity between MCF10A-*ras* (white) and MCF10CA1a (gold). PC activity is indicated by the M+3 labeling pattern of citrate following ^13^C-glucose incubation (right panel). **F** Pyruvate dehydrogenase (PDH) activity between MCF10A-*ras* (white) and MCF10CA11a (gold). PDH activity is indicated by the M+2 labeling pattern of citrate following ^13^C-glucose incubation (right panel). **G** Carbon flux from glutamine through the reverse tricarboxylic acid (TCA) cycle is indicated by the M+5 labeling pattern of citrate following ^13^C-glutamine incubation. **H** Schematic of substrates (gold) that contribute to synthesis of FAs within the MCF10CA1a cell. Asterisks indicate p < 0.05 between MCF10A-*ras* and MCF10CA1a among 3 or 4 biological replicates, as indicated by individual data points. *Abbreviations*: OAA = oxaloacetate; TCA = tricarboxylic acid cycle; DGAT = diacylglycerol acyltransferase; TAG = triacylglycerol.

We next sought to determine if FA synthesis contributes to the higher lipid accumulation in metastatic cells. To this end, we examined expression of key enzymes involved FA synthesis and found that ATP citrate lyase (ACLY) and FASN were significantly higher in the metastatic compared to non-metastatic cells (Fig. 3A and B). Additionally, MCF10CA1a cells had a lower total citrate pool size compared to MCF10A-*ras* cells (Fig. 3C). These data suggest that citrate was being depleted at faster rate in the MCF10CA1a cells, potentially as substrate for endogenous FA synthesis. Furthermore, incorporation of carbon from ^13^C-subtrates including acetate, glucose, and glutamine into palmitate was higher in the MCF10CA1a cells compared to MCF10A-*ras* cells (Fig. 3D). These results demonstrate a greater level of *de novo* lipogenesis in the metastatic compared to non-metastatic cells.

We next interrogated the activity of other enzymes related to substrate availability for FA synthesis. The labeling pattern of ^13^C from ^13^C_6_-glucose into citrate indicated higher pyruvate carboxylase (PC) and pyruvate dehydrogenase (PDH) activity in the metastatic compared to non-metastatic cells (Fig. 3E and F). Similarly, the percentage of M + 5 citrate labeling pattern from ^13^C_5_-glutamine in MCF10CA1a was significantly greater than that in the MCF10A-*ras* cells, demonstrating a higher level of reductive carboxylation of glutamine through the reverse TCA cycle (Fig. 3G–H). Together, these results indicate greater FA synthesis in metastatic compared to non-metastatic cells.

### Inhibition of Fatty Acid Synthase (FASN) decreases TAG stores and limits cell migration

**Figure 4:**
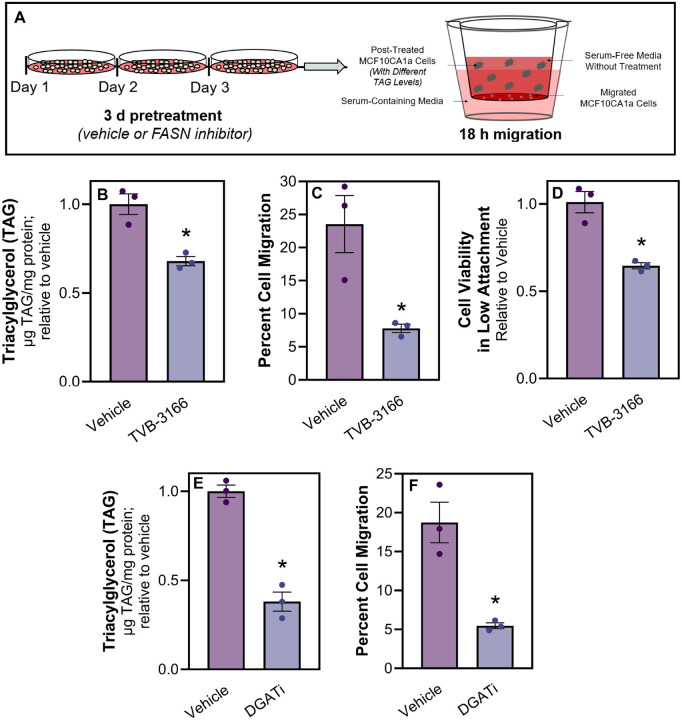
Inhibition of Fatty Acid Synthase (FASN) decreases TAG stores and limits metastatic cell migration. **A** Schematic of experimental design of migration assays. MCF10CA1a cells were treated with inhibitors denoted in each figure panel, or vehicle (DMSO). After 72 hours of treatment, TAG analysis was assessed or cells were plated for an additional 18 hours in a transwell, without any treatments present. **B** TAG and **C** migration were measured between vehicle (white) or 20 μM TVB-3166 (FASNi; grey) treated MCF10CA1a cells. **D** MCF10CA1a cell viability in detached conditions following vehicle (white) or TVB3166 (FASNi; grey) treatment for 72 hours. **E** TAG and **F** migration were measured between vehicle or simultaneous PF 04620110 + PF 06424439 (DGAT 1 and 2 inhibitors, respectively; DGATi; grey) treated MCF10CA1a cells. Asterisk indicates p < 0.05 between vehicle and inhibitor treated MCF10CA1a cells of 3 biological replicates.

Next, we utilized TVB-3166, a reversible inhibitor of FASN, to determine if FA synthesis contributes to TAG stores in metastatic cells (Fig. 4A). Inhibition of FASN significantly reduced TAG accumulation in the MCF10CA1a cells (Fig. 4B). Furthermore, this inhibition of FASN-derived TAG reduced migration of the MCF10CA1a cells (Fig. 4C). Additionally, viability of metastatic cells following culture in low attachment conditions was also reduced following TVB-3166-induced TAG reduction (Fig. 4D). We also reduced TAG stores in the MCF10CA1a cells via simultaneous inhibition of DGAT1 and DGAT2, the enzymes responsible for TAG synthesis. Similar to inhibition of FASN by TVB-3166, blockade of DGAT1 and 2 also reduced TAG accumulation and subsequent cell migration (Fig. 4E and F). Together, these results suggest that FASN-derived TAG is necessary for MCF10CA1a cell migration.

### FASN inhibition alters whole cell protein abundance patterns

**Figure 5:**
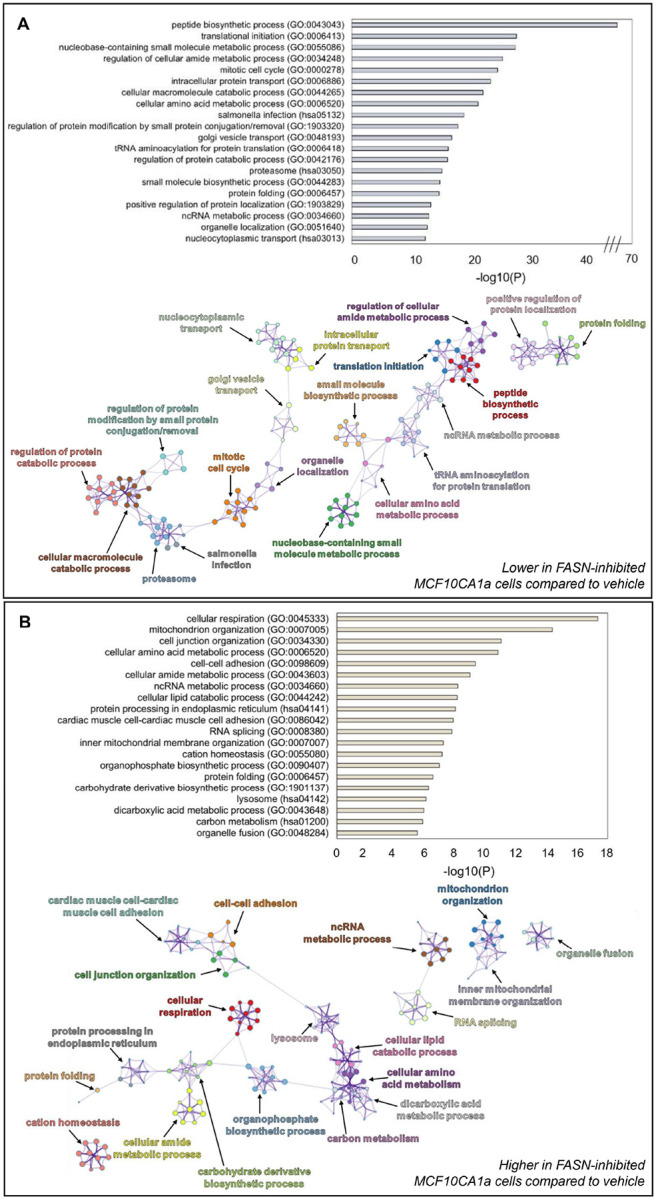
FASN-inhibition by TVB-3166 alters whole cell protein abundance patterns. **A** Top 20 clusters of statistically enriched Kyoto Encyclopedia of Genes and Genomes (KEGG) pathways and Gene Ontology Biological Processes (GO_BP) terms lower in FASN-inhibited MCF10CA1a cells compared to vehicle-treated cells (top). Enriched clusters of KEGG pathways and GO_BP terms higher in FASN-inhibited presented in network format (bottom). **B** Top 20 clusters of statistically enriched Kyoto Encyclopedia of Genes and Genomes (KEGG) pathways and Gene Ontology Biological Processes (GO_BP) terms higher in FASN-inhibited MCF10CA1a cells (top). Enriched clusters of KEGG pathways and GO_BP terms higher in FASN-inhibited presented in network format (bottom). The greater the −log10(P) value for top panels indicate more enriched terms. Statistically enriched similar terms are organized into clusters and colored based on the representative parent term for the cluster. Each circle within a colored cluster represents one term, and the size of the circle correlates with the number of proteins identified within that term. Similar terms are connected by a line, with a thicker line indicating higher similarly between terms. Enrichment values and cluster networks were calculated using Metascape.

To explore potential pathways that FASN inhibition might contribute to the reduced migratory and viability capacities of metastatic cells, we employed an untargeted proteomic analysis. Similar to the approach in Fig. 4, MCF10CA1a cells were treated with the FASN inhibitor TVB-3166 for 72 hours and total protein content was analyzed by mass spectrometry. Although similar numbers of proteins were identified in each condition (Fig. S1), 481 proteins were significantly higher and 805 proteins were significantly lower following TVB-3166 treatment. Proteins that did not change upon TVB-3166 treatment included those involved in ribosome and mRNA biogenesis/processing, protein transport and processing, and organelle organization (Fig. S2). Proteins present at significantly lower levels upon TVB3166 treatment or only identified in vehicle-treated cells were enriched in pathways involving protein catabolism, proteasome function, mitotic cell cycle, and macromolecule metabolism (Fig. 5A). Of note, ten of the top twenty protein categories that were reduced in TVB-3166-treated cells are involved in amino acid/protein metabolism. Proteins present at significantly higher levels of abundance or identified only after TVB-3166 treatment were enriched in pathways involving cellular respiration, mitochondrial organization, lipid catabolic processes, cell-cell adhesion, lysosome, and carbon metabolism (Fig. 5B). Enzymes comprising lipid metabolism were also enriched upon TVB-3166 treatment, including Acetyl-CoA Acetyltransferase (ACAT1), Electron Transfer Flavoprotein Subunit Alpha (ETFA), Propionyl-CoA Carboxylase Beta Chain (PCCB) and Acid Ceramidase Subunit Alpha (ASAH1) (Fig. S3). Other major metabolism-regulating proteins, including the aerobic glycolysis-inducer Forkhead Box Protein K1 (FOXK1), were only present after TVB-3166 treatment. Cell-cell adhesion proteins including cadherins and integrins were also more enriched following treatment with TVB-3166 (Fig. S4). Overall, these data present a global view of protein expression that is dependent upon FASN activity in metastatic cells.

### FASN is necessary for lung metastases

**Figure 6:**
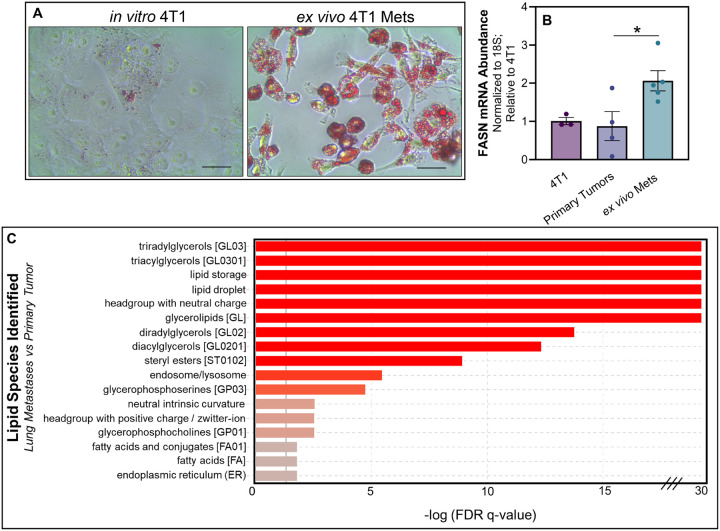
FASN expression and lipid accumulation are increased following metastasis. **A** Oil Red O staining of *in vitro* cultured 4T1 cells and those isolated from lung metastases. **B** Quantitative RT-PCR analyses of FASN expression in *in vitro* cultured 4T1 cells, or those cultured ex-vivo within 3 passages post isolation from either mammary fat pad primary tumors (1° tumors) or pulmonary metastases (mets). Data are normalized to FASN levels in *in vitro* cultured 4T1 cells and derived from four separate primary tumors and five separate pulmonary metastases. Asterisk indicates p < 0.05 between primary tumors and pulmonary metastases. **C** LIPID Informatics Analysis (LION) enrichment analysis comparing lipidomic pro les between *ex vivo* cultures of 4T1 cells from lung metastasis (LM) cells and primary tumor (PT). Lipid classes are ranked based on the -log of the q-value’s false discovery rate (FDR), with a higher value indicating greater enrichment. Red bars denote lipid classes that are significantly more enriched in LM cells compared to PT cells, while grey bars represent lipid classes with no significant difference in enrichment.

**Figure 7:**
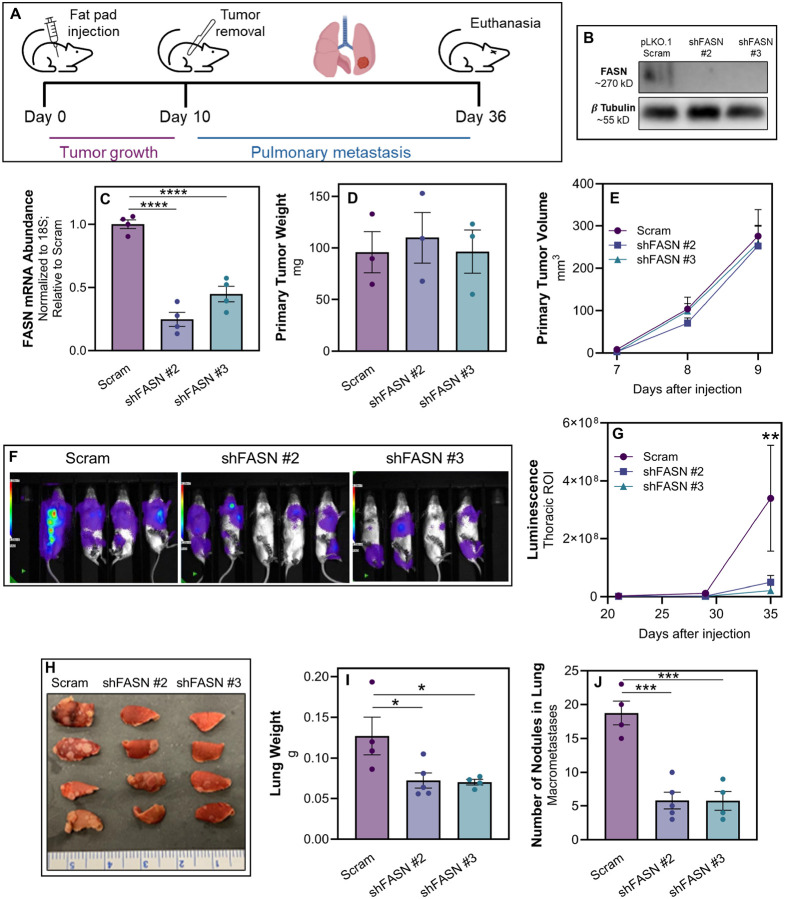
FASN is required for lung metastasis. **A** Schematic representation of the experimental design for assessing the impact of FASN depletion on tumor progression and metastasis in 4T1 cells. Following fat pad engraftment of 4T1 cells, primary tumors were surgically removed and subsequent development of pulmonary metastasis was monitored by bioluminescence over 26 days. **B** Western blot analysis demonstrating depletion efficiency of FASN in 4T1 cells expressing a scrambled shRNA (pLKO.1 scram) or shRNAs targeting FASN (shFASN #2 and #3). β-Tubulin served as a loading control. **C** Quantitative RTPCR analysis of FASN mRNA expression levels in 4T1 cells post-transfection with scrambled shRNA or shFASN constructs. Data represents mRNA expression fold change, normalized to GAPDH expression. Data are the mean of 3 biological replicates completed in triplicate where **** indicates p < 0.0001. **D** Bar graph showing the weight of 4T1 primary tumors upon excision from the mammary fat pad. The weights are presented as mean ± SEM. **E** Growth curve representing the volume of mammary tumors measured at the indicated time points following fat pad engraftment. **F** *In vivo* bioluminescent imaging of mice bearing control (Scram) and FASN-depleted (shFASN) 4T1 metastases, 35 days after fat pad engraftment. **G** Quantitative analysis of thoracic bioluminescence from 4T1 tumor bearing mice. Data represent the mean thoracic luminescence intensity ± SEM for each group at specified time points after cell injection. **H** Photograph of excised lungs from mice in each treatment group, showing differences in macroscopic metastases burden. **I** Bar graph depicting the total lung weights from each group. Weights are presented as mean ± SEM. **J** Quantification of macroscopic nodules present in the lungs. For panels D-J, data are the mean of each group (n ≥ 4) ± SEM, with ‘ns’ indicating no significant difference, * indicating p < 0.05, and *** indicating p < 0.001.

We next sought to expand on our observations to additional models of metastasis. First we analyzed EGFR-transformed murine mammary gland cells (NME) as compared to their isogenic counterparts harvested from lung metastases (NME-LM2) (33). Consistent with our findings in the MCF10A system, enhanced lipid accumulation was observed in the LM2 cells, and this could be mitigated by TVB-3166 (Fig. S5). Additionally, we have previously demonstrated the cellular plasticity of the 4T1 model as these cells form a primary tumor and progress to pulmonary metastases (43). As such, 4T1 cells were engrafted onto the mammary fat pad and allowed to metastasize to the lungs. Upon necropsy, *ex vivo* 4T1 cultures were established from primary tumors and pulmonary metastases. This *ex vivo* approach similarly demonstrated that freshly isolated metastatic cells had increased lipid accumulation and FASN expression (Fig. 6A and B). To further characterize lipid content in metastases, we conducted total lipidomic analyses of 4T1 *ex vivo* cultures derived from primary tumors and lung metastases. This approach similarly demonstrated that freshly isolated, *ex vivo* lung metastases had significantly greater levels of LD-associated neutral lipids, such as TAG, compared to *ex vivo* primary tumors harvested from the same animals (Fig. 6C). To determine whether FASN is required for lung metastasis, we depleted its expression in the 4T1 cells using two independent shRNAs, and these cells were engrafted onto the mammary fat pad of the BALB/c mice (Fig. 7A–C). No differences in primary tumor growth were observed upon FASN depletion compared to control (Fig. 7D and E). However, FASN-depletion significantly reduced metastasis as measured by bioluminescence, total lung weight, and gross enumeration of macroscopic metastases (Fig. 7F–J).

## Discussion

Through our assessment of metabolic differences between metastatic and non-metastatic breast cancer models, we observed elevated lipid accumulation to be associated with breast cancer metastasis. Therefore, we sought to define pathways contributing to LD storage and determine their role in promoting breast cancer progression. We found that metastatic cells have significantly higher levels of *de novo* lipogenesis and that these FASN-derived TAG stores are necessary to sustain FAO-dependent cell migration, a key step necessary for metastasis. Interestingly, the non-metastatic cells, which contain low levels of TAG, do not rely on FAO for migration. While both cell lines have similar levels of palmitate uptake, the non-metastatic MCF10A-*ras* cells have a significantly higher rate of exogenous palmitate oxidation compared to the metastatic MCF10CA1a cells. This suggests that the metastatic cells store FAs that they internalize prior to oxidizing them, or that these extracellular FAs are utilized for non-oxidative functions. Notably, when no FAs were supplied in the media, the MCF10CA1a cells had significantly higher oxygen consumption from FAO than the non-metastatic cells, supporting the concept that the lipid stores within metastatic cells are catabolized for subsequent oxidative phosphorylation. In sum, these experiments demonstrate the necessary role of TAG stores in promoting metastatic breast cancer migration, and that the higher level of stored TAG requires upregulated FASN activity. We have established that FA metabolism within metastatic cells is highly dysregulated, where both FA synthesis and catabolism likely occur simultaneously. Dual activation of these pathways does not occur under healthy circumstances, suggesting that concurrent identification of FA synthesis and catabolism could be a powerful predictor of metastasis.

Our results establish that FASN-derived TAG is necessary not only for the migration of MCF10CA1a cells, but also for their survival in detached conditions, two processes required for breast cancer metastasis. Using untargeted global proteomic analyses, we define several proteins and pathways that require FASN activity in metastatic cells. For example, inhibition of FASN increased cell-cell adhesion-related proteins compared to vehicle-treated cells, consistent with the requirement for cell-adhesion to maintain appropriate cell attachments to other cells and the extracellular matrix. These findings complement other studies that have previously established a connection between lipid metabolism and epithelial-mesenchymal transition (44–48). Furthermore, FA catabolism and processing enzymes, as well as other major metabolism regulators such as FOXK1, were more enriched upon FASN inhibition. These findings lay the groundwork for further understanding of how FA synthesis promotes breast cancer invasion, dissemination, and metastasis.

The functional importance of FASN was substantiated using the 4T1 model. FASN levels and lipid accumulation significantly increased following metastasis to the lung. Collectively, these results suggest that FASN plays an important role in successful completion of various steps of the metastatic cascade, including migration, cell survival, and adaptation. The mechanisms that regulate FASN expression and/or activity during completion of the metastatic cascade remain to be elucidated. Nonetheless, our findings add to a growing body of evidence indicating that FASN expression is enhanced upon metastatic progression (20, 49–51). A recent study demonstrated that FASN is required for brain metastasis, and suggests that the requirement of FASN-derived lipids is unique to that site given the lack of FA availability from the brain’s microenvironment (52). Our results indicate a role of *de novo* lipogenesis for robust breast to lung metastasis as well. A potential explanation for these results is that our studies are derived from the 4T1 model, which allows for assessment of primary tumor growth and other aspects of the metastatic cascade following primary tumor removal. This raises the interesting possibility that aspects of the primary tumor such as hypoxia, cytokine, or immune exposure drive differential FASN expression and lipid metabolism in subsequent metastases.

In summary, our results indicate that FA synthesis provides lipids for storage in LDs, and that these FAs may be oxidized during stress to support metastatic breast cancer cell migration, survival, and overall metastasis. In addition to the role of stored TAG accumulation from FA synthesis, other potential proteins, such as those involved in cell-cell adhesion, are differentially regulated following FASN-inhibition. These events likely act in concert to contribute to the anti-cancer effects observed upon FASN inhibition.

## Conclusions

The results reported here provide insight into how dysregulated lipid metabolism in metastatic breast cancer cells contributes to specific steps in metastasis, and thus highlight proteins such as FASN that could be targeted to minimize the negative effects of lipid-rich, metastatic breast cancer progression.

## Figures and Tables

**Table 1:** Primers used in qPCR analysis of gene expression. Written in 5’◊3’ direction.

Gene name and species	Forward Primer	Reverse Primer
18S, H	ATCCCTGAGAAGTTCCAGCA	CCTCTTGGTGAGGTCGATGT
MAGL, H	GTCAATGCAGACGGACAGTA	AGAACCAGAGGCGAAATGAG
CPT1a, H	ATCAATCGGACTCTGGAAACGG	TCAGGGAGTAGCGCATGGT
CPT1b, H	GCGCCCCTTGTTGGATGAT	CCACCATGACTTGAGCACCAG
ACSL1, H	CTTATGGGCTTCGGAGCTTTT	CAAGTAGTGCGGATCTTCGTG
ACLY, H	GAAGGGAGTGACCATCATCG	TTAAAGCACCCAGGCTTGAT
GAPDH, M	CAACTTTGGCATTGTGGAAGGGCTC	GCAGGGATGATGTCTGGGCAGC
FASN, M	AGAGATCCCGAGACGCTTCT	GCTTGGTCCTTTGAAGTCGAAGA

*Abbreviations*: H = human, M = murine, MAGL = monoacylglycerol lipase, CPT = carnitine palmitoyltransferase, ACSL = long-chain fatty acyl-CoA synthetase, ACLY = ATP citrate lyase, GAPDH = glyceraldehyde-3-phosphate dehydrogenase, FASN = fatty acid synthase.

## Data Availability

Raw LC-MS/MS data is available on the Mass Spectrometry Interactive Virtual Environment (MassIVE) data repository at: ftp://massive.ucsd.edu/v08/MSV000095941/. All other raw data are available upon request from the corresponding author.

## Abbreviations

ACLY: ATP Citrate Lyase
ACC: Acetyl-CoA Carboxylase
AUC: area under the curve
ACAT1: Acetyl-CoA Acetyltransferase
ASAH1: Acid Ceramidase Subunit Alpha
BCA: bicinchoninic acid assay
CPT1: Carnitine Palmitoyltransferase
DGAT: Diacylglycerol Acyltransferase
DMSO: dimethyl sulfoxide
DPM: disintegrations per minute
ECM: extracellular matrix
ETFA: Electron Transfer Flavoprotein Subunit Alpha
FA: fatty acid
FASN: Fatty Acid Synthase
FAO: fatty acid oxidation
FDR: false discovery rate
GO_BP: Gene Ontology Biological Process
KEGG: Kyoto Encyclopedia of Genes and Genomes
LD: lipid droplet
LM: lung metastasis
MTT: 3–4,5-dimethylthiazol-2-yl-2,5-diphenyltetrazolium bromide
MRM: multiple reaction monitoring
OCR: oxygen consumption rate
PMSF: phenylmethanesulfonyl fluoride protease inhibitor
PC: Pyruvate Carboxylase
PDH: Pyruvate Dehydrogenase
PCCB: Propionyl-CoA Carboxylase Beta Chain
PT: primary tumor
qPCR: quantitative polymerase chain reaction
RIPA: radioimmunoprecipitation assay
TCA: tricarboxylic acid
TNBC: triple negative breast cancer
TAG: triacylglycerol
TEM: transmission electron microscopy
WCL: whole cell lysate
